# A Framework for the Economic Analysis of Data Collection Methods for Vital Statistics

**DOI:** 10.1371/journal.pone.0106234

**Published:** 2014-08-29

**Authors:** Eliana Jimenez-Soto, Andrew Hodge, Kim-Huong Nguyen, Zoe Dettrick, Alan D. Lopez

**Affiliations:** 1 School of Population Health, The University of Queensland, Australia; 2 Centre for Applied Health Economics, School of Medicine, Griffith University, Australia; 3 School of Population and Global Health, The University of Melbourne, Australia; US Army Engineer Research and Development Center, United States of America

## Abstract

**Background:**

Over recent years there has been a strong movement towards the improvement of vital statistics and other types of health data that inform evidence-based policies. Collecting such data is not cost free. To date there is no systematic framework to guide investment decisions on methods of data collection for vital statistics or health information in general. We developed a framework to systematically assess the comparative costs and outcomes/benefits of the various data methods for collecting vital statistics.

**Methodology:**

The proposed framework is four-pronged and utilises two major economic approaches to systematically assess the available data collection methods: cost-effectiveness analysis and efficiency analysis. We built a stylised example of a hypothetical low-income country to perform a simulation exercise in order to illustrate an application of the framework.

**Findings:**

Using simulated data, the results from the stylised example show that the rankings of the data collection methods are not affected by the use of either cost-effectiveness or efficiency analysis. However, the rankings are affected by how quantities are measured.

**Conclusion:**

There have been several calls for global improvements in collecting useable data, including vital statistics, from health information systems to inform public health policies. Ours is the first study that proposes a systematic framework to assist countries undertake an economic evaluation of DCMs. Despite numerous challenges, we demonstrate that a systematic assessment of outputs and costs of DCMs is not only necessary, but also feasible. The proposed framework is general enough to be easily extended to other areas of health information.

## Introduction

Health information, in particular vital statistics and cause-of-death (COD) data, is an essential public good. However, every year, one-third of births are not officially recorded, and three-quarters of all deaths are either not registered or lack a medically certified cause [1]. Recent global initiatives have thus called for significant improvements in country systems to register births, deaths and causes of death [2], [3].

The preferred data collection method (DCM) for vital statistics is a complete system of civil registration for vital statistics (CRVS) with all deaths assigned an underlying cause by a medically qualified doctor according to the International Classification of Diseases (ICD) [4]. In addition to providing essential data on vital statistics, civil registration also serves important public functions, which has significant implications for human rights [2]. Citizens benefit directly by having a birth certificate which recognises their existence and legal identity, and this allows them access to a range of health and social services such as schooling, immunisation programs, subsidised housing, old age care and passports. In effect, it enables them to participate fully in society and to enjoy societal benefits [5]. By registering deaths, families of the deceased can gain access to insurance/inheritance benefits.

Notwithstanding the well documented social benefits of CRVS [2], [6], [7], very few low- and middle-income countries are making substantive progress in advancing this agenda [6], [8]. If they are complete and accurate they will contain important information on the age and cause of death, and on age of mother, birth spacing/parity and place of residence for births. When compiled, and used, these statistics are a powerful source of information to inform and guide health and social policies [9] to prevent premature deaths, alert health policy and health services to the need for rapid responses to emerging epidemics (such as HIV), or established avoidable causes of mortality such as alcohol use and traffic fatalities. They also provide important information about the nature, extent and trends in health inequalities in populations and the main diseases and injuries that underlie them, and serve to guide the research community about knowledge gaps relating to the epidemiology of diseases or injuries causing greatest burden and for which information about cost-effective interventions is lacking [2]. Vital statistics on births provide similarly important information support for health and social policies to improve the health, well-being and survival of mothers and children. However, reaching the goal of complete systems of CRVS in a timely fashion, especially in developing countries, will be extremely difficult. The number of countries reporting the registration of more than 90 per cent of all in-country deaths using CRVS increased by only seven from the 1970s to the 1990s [2]. Most continue to invest in interim measures, such as surveys, to obtain mortality rates and cause-of-death distributions. However, the decision to invest scarce resources into particular DCMs is often made without even a basic understanding of their relative costs and benefits.

To date there have been no documented attempts to undertake a formal comparative assessment of the costs and outcomes/benefits of the various methods available to collect vital statistics. In this paper, we propose a systematic framework for such a comparative assessment. We test the framework with simulated data of a stylised scenario in a hypothetical low-income country.

## Methods

### The systematic framework

**Figure 1:**
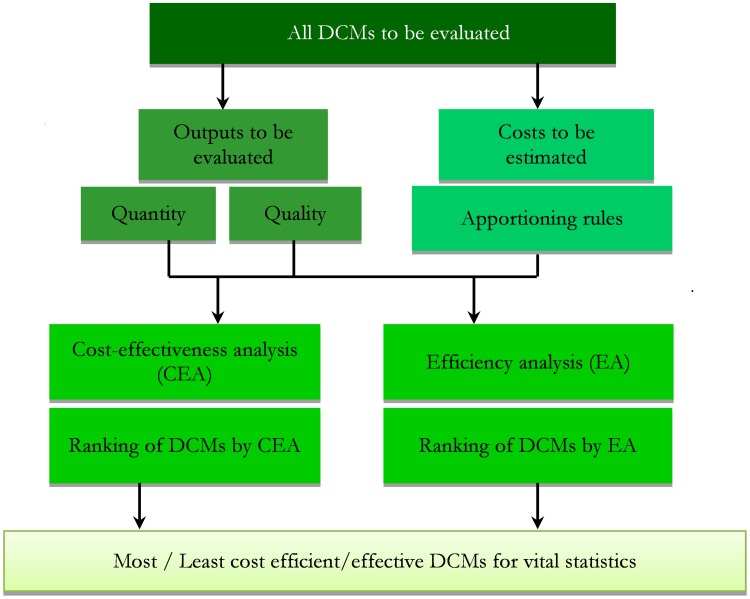
Systematic assessment of data collection methods (DCMs) – the economics approach.

The proposed framework represented in Figure 1 comprises of four elements. The first three elements form the ‘context assessment’, where the necessary information to perform the economic analysis is collated. The fourth element, the ‘quantitative assessment’, proposes two economic tools to systematically assess the comparative costs and outcomes/benefits of the various alternatives. To the authors' knowledge, there is no previously documented attempt to construct such a framework to examine and ultimately determine the most efficient method to collect vital statistics data; that is, the optimal mixture of quality-quantity and cost. Further details are provided in File S1.

#### Element 1: Identify the alternatives to be evaluated

A literature search to identify the various alternatives to be evaluated forms the first element of the proposed framework. In our case, using a combination of the MESH terms ‘vital statistics’ and ‘data collection’, we searched the MEDLINE and SCOPUS databases to identify the methods to collect data on vital events when complete CRVS is not available. A total of 256 references published since 1970 were found, and to ensure all relevant papers were included, we checked the reference lists of selected articles. As summarized in Table 1, in addition to CRVS we identified 8 DCMs, which were classified into three major categories: partial registration; censuses and population surveys; and facility-based data collection.

**Table 1:** Data collection methods for vital statistics.

System Type	DCM	Description
Complete or partial registration systems	Civil registration	Births and deaths in the population are continuously recorded.
		Deaths are recorded with a medically certified COD.
		Yearly statistics are generated based on this data.
	Sample registration	Births and deaths in a representative sample of the population are continuously recorded.
		In some systems, deaths in the sample are recorded with MCOD.
		In other cases, deaths in the sample are recorded with a COD assigned using VA. Depending on the method, the resulting data may be grouped either by broad or specific causes of death.
		Yearly statistics are generated based on this data.
	Demographic surveillance sites	Births and deaths in a non-representative sample of the population are continuously recorded.
		Deaths in the sample are recorded with a COD assigned using VA. Depending on the method, the resulting data may be grouped either by broad or specific causes of death.
		Yearly statistics are generated based on this data.
Census and surveys	Population census	All households are queried regarding current occupants, as well as details of recent births and deaths.
		For a system with full VA; that is, a COD exists for each recorded death, a VA questionnaire is used to assign a COD.
		For a system with partial VA COD, for a representative sample of recorded deaths, a VA questionnaire is used to assign a COD.
		Alternatively COD distribution may be generated through modelling based on age–sex patterns, prevalence of risk factors and intervention coverage.
		Statistics are usually generated every 10 years.
	National-level household survey: direct estimates	A representative sample of households is queried regarding current occupants, as well as details of recent births and deaths.
		For a survey using VA COD, for each recorded death a VA questionnaire is used to assign a COD.
		Alternatively, COD distribution may also be generated through modelling based on age–sex patterns, prevalence of risk factors and intervention coverage. Statistics are usually generated every three to five years.
	National-level household survey: indirect estimates	A representative sample of households is queried regarding current occupants, as well as survival status of siblings and/or children.
		COD distribution is generated through modelling based on age–sex patterns, prevalence of risk factors and intervention coverage.
		Statistics are usually generated every three to five years.
	Sub-national-level household survey	A sample of households is queried regarding current occupants, as well as details of recent births and deaths.
		For each recorded death, a VA questionnaire is used to assign a COD.
		Statistics are usually generated every three to five years.
Facility-based collection	Facility-based reporting: wide scale	Births and deaths that occur within medical facilities are continuously recorded.
		Deaths are recorded with MCOD.
		Yearly statistics are generated based on this data.
	Facility-based reporting: sentinel sites	Births and deaths that occur within a representative subset of medical facilities are continuously recorded.
		Deaths are recorded with MCOD.
		Yearly statistics are generated based on this data.

*Notes*: DMC, data collection method; MCOD, medical certification of death; VA, verbal autopsy; COD, cause-of-death.

At first glance, all DCMs should be included in the evaluation. However, the government policy in regards to CRVS should dictate whether it is treated as the gold standard or as one of the alternatives to be evaluated. For some governments, the long-term policy might be to work towards the implementation of a complete CRVS (i.e. due to the associated social benefits, including human rights). CRVS is thus viewed as a policy with inherent social value and the focus of the evaluation is on the interim measures required to provide vital statistics data while the system is put in place. This perspective also conforms to the view that the main role of CRVS is not to provide data, but to serve important public functions such as the establishment of legal identities, which can facilitate the provision of social services. In this case, CRVS should be excluded from the evaluation as we propose in Scenario A below. On the other hand, if the government has no long-term policy of implementing CRVS, it is then included as one of the DCMs to be evaluated as in Scenario B. This is line with the view that CRVS is just another DCM and any additional social benefits are just externalities. As outlined below, this distinction also has significant implications for the choice of quantity measurement, and ultimately, the results of the evaluation.

#### Element 2: Identify and measure the outcomes of the alternatives to be evaluated

The second element of the proposed framework involves quantifying the outcomes of the various alternatives, which in some cases may be intangible. Any proposed metric of DCMs' outcomes will need to capture both the quantity and quality dimensions of data. There is no clear guidance regarding the appropriate units to measure the quantity outcomes produced by DCMs. Instead, we suggest that the choice be dependent on the government's long-term policy related to its CRVS. In the instance of a long-term policy to establish CRVS, the government would seek to collect all unit records of vital statistics for a country's population. The ‘quantity of data’ should thus be defined as the ‘unit records collected by each DCM’, approximated by the sample size of the DCM. On the other hand, if the establishment of CRVS is not part of the government's policy, a metric such as the ‘number of people represented by each DCM’ might be more suitable and in line with the need to produce vital statistics that are representative of the population.

Assessing the quality of each DCM is equally difficult and we utilised the assessment framework of Mahapatra *et al.* [2], which expanded the first framework evaluating quality of mortality and COD data proposed by Ruzicka and Lopez [10]. The framework has five quality attributes and we developed criteria for each category as detailed in Table 2. To operationalize this framework, two issues must be addressed. First, the government's policy on CRVS will again need to be taken into account. When the government seeks to establish a complete CRVS, the criteria used to evaluate the interim methods should include the extent to which they enable or undermine such a policy. Therefore, ‘improvement towards CRVS’ is added as one of the quality criteria. Second, an appropriate (albeit indicative) ordinal scale must be assigned under each criterion to provide a numeric measure of quality. We implemented a straightforward approach and assumed a basic linear scoring system with a range of [0–10] applied to each quality criteria (see the File S1 for further discussion on potential scoring systems).

**Table 2:** Assessment framework for vital statistics.

Criteria	General vital statistics	Cause-of-death statistics
**Accuracy (A)**		
	A1- Accuracy of vital event statistics	A2 - Accuracy of cause-of-death statistics
Coverage	% of population living in areas where vital event recording occurs	% of population living in areas where COD recording occurs
Completeness	% of events contributing to fertility/mortality statistics	% of deaths with appropriately certified COD
Missing data	% of key variables with response not stated	% of COD reports for which age–sex data are missing
Use of ill-defined categories	N/A	% of deaths classified under various miscellaneous and ill-defined categories
Improbable classifications	N/A	Number of deaths assigned to improbable age or sex categories per 100 000 coded deaths
Consistency between cause of death and general mortality	N/A	% of COD data points deviating more than 2 (or 3) SDs from general mortality-based predictions
**Relevance (R)**		
	R1- Relevance of vital event statistics	R2 - Relevance of cause-of-death statistics
Routine tabulations	By sex and five-year age groups, based on place of usual residence. Deaths in children under five years tabulated by 0 and 1–4 year age group.	By sex, and at least by eight broad age groups; namely, 0, 1–4, 5–14, 15–29, 30–44, 45–59, 60–69 and 70+ years
Small-area statistics	Number of vital event tabulation areas per million population	Number of COD tabulation areas per million population
**Comparability (C)**		
	C1- Comparability of vital event statistics	C2 - Comparability of cause-of-death statistics
Over time	Stability of key definitions over time	Consistency in the proportions of cause-specific mortality over consecutive years
Across space	Uniformity of definitions across areas	ICD to certify and code deaths, revision used and code level to which tabulations are published
**Timeliness (T)**		
	T1 - Timeliness of vital event statistics	T2 - Timeliness of cause-of-death statistics
Production time	Mean time from end of reference period to publication	Mean time from end of reference period to publication
Regularity	SD of production time	SD of production time
**Accessibility (AC)**		
	AC1 - Accessibility of vital event statistics	AC2 - Accessibility of cause-of-death statistics
Media	Number of formats in which data are released	Number of formats in which data are released
Metadata	Availability and quality of documentation	Availability and quality of documentation
User service	Availability and responsiveness of user service	Availability and responsiveness of user service

*Notes:* Adapted from Table 1, in Mahapatra *et al*. [2] (p. 1654). SD, standard deviation; ICD, International Classification of Diseases; COD, cause-of-death; N/A, not applicable.

#### Element 3: Identify and measure the costs of the alternatives to be evaluated

A comparative assessment of the costs of using the alternatives forms the third element of the proposed framework. While there is a vast literature on the methodologies for costing exercises, particularly in health services [11], the costs with respect to DCMs are not well documented. The absence of costing data and appropriate costing frameworks for DCM have been identified as a hindrance for development partners and governments trying to make informed decisions on investments in DCM [12]. In File S1 we briefly describe the major issues involved in assessing the costs of DCMs and the modelling assumptions that we have used for this study.

#### Element 4: An economic assessment of the alternatives to be evaluated

Using the information collated in elements 1 to 3, the final element of the framework involves the use of cost-effectiveness analysis (CEA) and efficiency analysis to quantitatively assess the comparative effectiveness and efficiency of the various alternatives with a view to inform investment decisions. Note that there are a number of conceptualisations of efficiency in the economics literature. In our context, we are referring to the methods and definitions used in the productivity and efficiency literature, which is built on production economics [4]. Efficiency in this literature is defined as the ability to achieve maximal outputs from a given set of inputs (see File S2 for further details).

To utilise CEA, we would need to aggregate the scores into a single index of quality that could then be used as a standardised metric of the DCMs outcomes. Table 3 presents a brief description, and the main advantages and disadvantages of alternative methods that can be used to produce a composite index. Given that no method is superior, we test the robustness of our results using multiple methods. The CEA methodology produces a ‘cost per quality-adjusted data index’ for each DCM and this ratio is used to rank the alternative DCMs. On the other hand, efficiency analysis does not require a composite quality index and produces a cost-efficiency index that can be used for ranking. This index has a range of [0–1] and the closer the index to unity, the more cost efficient the DCM.

**Table 3:** Alternative methods for aggregating the quality scores into a single index.

Methods	Description	Disadvantages	Advantages
Unweighted average	Sum of all scores divided by the total number of criteria	Assumes all quality attributes are equally important in all circumstances and all settings	Calculation simplicity
			Does not require a priori knowledge about the relative importance of different quality criteria
			Aggregation index for individual
			DCM is independent from the rest
Weighted average – by expert opinion	Weights assigned to each criterion are identified a priori by experts.	The weights are subjective and expert consensus might be difficult to achieve.	Calculation simplicity
			Reflects the relative importance of different quality criteria, which might vary by setting
DEA-based aggregation	Weights are assigned via an optimisation problem that includes all DCMs to be evaluated. Used in several applications of multi-criteria decision analysis (MCDA) in a wide range of policy areas, including health.	Requires a large number of observations. If only a handful of DCMs are evaluated, it would require several assessments (i.e. by various experts) of each DCM.	Does not require a priori knowledge about the relative importance of the quality criteria.
			Data driven
			No systematic bias toward any quality criteria. Might be useful when there are conflicting opinions on the relative importance of each quality criteria

*Notes*: DEA, Data Envelopment Analysis; DMC, data collection method.

### Simulated stylised example

Given the limited availability of data, particularly with respect to costings, we relied on simulated data to demonstrate how the systematic assessment framework can be applied. The stylised example assumes a hypothetical low-income country with a population of 33 million which has developed DCMs of varying quality operating in parallel. Our hypothetical country has an established CRVS but it remains incomplete. Nine other health and demographic information systems collecting vital statistics data exist – note that when undertaking this exercise, the country should organise a panel of experts to discuss the feasibility of the evaluation, map all the potential DCMs and then identify those with a primary or secondary objective to collect vital statistics data. Each system is briefly described in Table 4. We assume that substantial investments have been made in those systems and policymakers are interested in assessing which DCMs are providing the best value for money. Given the importance within the framework of the government's policy to either establish a civil registration system in the long-term, we consider two scenarios (A and B) assuming alternatively one of the two options. Table 5 describes the resultant differences between the two scenarios.

**Table 4:** Stylised Example: Health information systems and their characteristics.

No.	Information system	Data collection methods for vital statistics used	Collection objectives	Area of coverage	Number of participants
1.	National housing and population census (census)	Population-based census: Long form survey	Demographic, poverty, housing, labour for participation and health indicators	Nationwide	33 000 000
2.	National Household Survey (NHS)	Household survey	Income and poverty focus, with some demographic and health indicators	Nationwide	23 000
3.	Demographic and Health Survey (DHS)	Household, community and facility based surveys	Mortality, fertility and use of maternal and child health service	Nationwide	15 000
4.	Vital registration (CRVS)	Population-based forms	Compulsory birth and death registration	>60% districts	3 000 000
5.	Health management system (HMS)	Facility-based forms	Continuous collection of morbidity, mortality, and service coverage	Health facility nationwide	17 000 000
6.	Integrated disease surveillance (IDS)	Facility-based forms	Continuous data collection on disease and mortality	Health facility nationwide	3 000 000
7.	Demographic surveillance system: Region X (X-DSS)	Population-based census: Mortality surveillance using verbal autopsy	Regular documentation of births, deaths, migrations and socioeconomic information	Some districts in Region X	66 000
8.	Demographic surveillance system: Region Y (Y-DSS)	Population-based census: Mortality surveillance using verbal autopsy	Regular documentation of births, deaths, and health service utilisation	Some districts in Region Y	83 000
9.	Adult morbidity and mortality project (MM)	Population-based census: Mortality surveillance using verbal autopsy	Regular collection of information on burden of disease and mortality	Some districts across the country	500 000
10.	Demographic surveillance system for AIDS (A-DSS)	Population-based census: Mortality surveillance using verbal autopsy; population-based HIV surveillance	HIV surveillance and some mortality data collection within the surveillance site	Villages within a specific region	23 000

*Notes*: No., number.

**Table 5:** Stylised Example: Description of alternative scenarios.

Assumption	Scenario A	Scenario B
Long-term policy of establishing a complete CRVS	Yes	No
(a) CRVS included in the list of DCMs to be evaluated	No	Yes
(b) Measure of ‘quantity of output’	Unit records*	Target population
(c) ‘Improvement toward CRVS’ included in the list of quality criteria	Yes	No
(d) Apportioning rule for costs of CRVS	N/A	30%

*Notes*: * Unit records are approximated using sample size. CRVS, civil registration for vital statistics; DMC, data collection method; N/A, not applicable.

When measuring the quality attributes of each DCM, we require a scoring matrix with the range of potential scores for each attribute. We have assumed a scoring panel of six individuals, providing both a consensus and individual scores. We use a random data generation process to provide the six individual scores, with a triangular distribution with predefined mean, minimum and maximum values within the range of [1]–[10]. Tables 6 and 7 present these hypothetical ‘consensus’ scores assigned to each DCM under each quality criteria, while the individual scores used are available in File S3. While the quality scores assigned to each DCM are hypothetical, they are based on assumptions of what might be plausible in such a hypothetical low-income country.

**Table 6:** Simulated data for Scenario A.

Data collection methods	Quality performance using consensus scoring matrix						Composite quality index				Costs		
	Accuracy	Relevance	Consistency	Timeliness	Accessibility	Improvement	Unweighted		DEA-based		Total cost	Apportion rule (%)	Cost for vital statistics
							Consensus matrix	Individual matrix	Consensus matrix	Individual matrix			
Census	2.33	8.00	5.50	3.00	5.67	4.00	0.7600	0.7786	1.0000	1.0000	$8 500 000	25	$2 125 000
NHS	0.83	2.50	4.50	3.00	6.67	2.00	0.5200	0.5401	0.8860	0.8843	$840 000	5	$42 000
DHS	1.00	4.00	6.00	5.00	7.67	2.00	0.6844	0.7195	1.0000	1.0000	$870 000	10	$87 000
HMS	4.83	2.50	5.00	7.00	3.67	8.00	0.8267	0.8760	1.0000	1.0000	$2 200 000	20	$440 000
IDS	3.17	1.00	5.50	9.00	4.00	6.00	0.7644	0.7137	1.0000	0.9773	$4 500 000	15	$675 000
X-DSS	5.00	6.50	4.50	7.50	4.67	7.00	0.9378	0.9122	1.0000	0.9958	$200 000	40	$80 000
Y-DSS	6.00	6.50	4.50	8.50	5.00	7.00	1.0000	1.0000	1.0000	1.0000	$220 000	40	$88 000
MM	4.50	5.00	5.50	7.50	4.00	6.00	0.8667	0.8760	1.0000	0.9932	$100 000	60	$60 000
A-DSS	5.00	4.50	5.50	8.50	5.00	5.00	0.8933	0.9561	1.0000	1.0000	$15 000	30	$4 500

*Notes*: See Table 4 for definitions and further details on the DCMs. DMC, data collection method; DEA, Data Envelopment Analysis.

**Table 7:** Simulated data for Scenario B.

Data collection methods		Quality performance using consensus scoring matrix					Composite quality index				Costs		
	Target population	Accuracy	Relevance	Consistency	Timeliness	Accessibility	Unweighted		DEA-based		Total cost	Apportion rule (%)	Cost for vital statistics
							Consensus matrix	Individual matrix	Consensus matrix	Individual matrix			
Census	33 000 000	2.33	8.00	5.50	3.00	5.67	0.8033	0.7776	1.0000	1.0000	$8 500 000	25	$2 125 000
NHS	33 000 000	0.83	2.50	4.50	3.00	6.67	0.5738	0.5471	0.8700	0.8682	$840 000	5	$42 000
DHS	33 000 000	1.00	4.00	6.00	5.00	7.67	0.7760	0.7335	1.0000	1.0000	$870 000	10	$87 000
CRVS	20 500 000	0.33	0.50	4.50	5.00	0.67	0.3607	0.3407	0.7740	0.8252	$750 000	30	$225 000
HMS	33 000 000	4.83	2.50	5.00	7.00	3.67	0.7541	0.8677	0.9360	0.9843	$2 200 000	20	$440 000
IDS	6 000 000	3.17	1.00	5.50	9.00	4.00	0.7432	0.7114	1.0000	0.9750	$4 500 000	15	$675 000
X-DSS	510 000	5.00	6.50	4.50	7.50	4.67	0.9235	0.9178	0.9700	0.9888	$200 000	40	$80 000
Y-DSS	180 000	6.00	6.50	4.50	8.50	5.00	1.0000	1.0000	1.0000	1.0000	$220 000	40	$88 000
MM	2 600 000	4.50	5.00	5.50	7.50	4.00	0.8689	0.8778	0.9980	0.9863	$100 000	60	$60 000
A-DSS	23 000	5.00	4.50	5.50	8.50	5.00	0.9344	0.9639	1.0000	1.0000	$15 000	30	$4 500

*Notes*: See Table 4 for definitions and further details on the DCMs. DMC, data collection method; DEA, Data Envelopment Analysis.

To plausibly approximate the total costs of various DCMs, we sourced information from the only available study on costing government information systems for social policy [13], [14]. The assumptions used in apportioning those costs although designed to be reasonable are entirely hypothetical. We applied some arbitrary apportioning rules to the total annual costs derived from the costing approximations [14]. It should be noted that some costs were underestimated since fixed cost was not included in the calculations for some DCMs. In these instances, we have apportioned a relatively larger percentage of costs to account for this potential underestimation of fixed cost.

### Ethics statement

Full review of this study from an institutional review board was not sought as this manuscript involved data analysis of simulated datasets, which are provided in File S3 and Tables 6 and 7 below.

## Results

Table 6 presents the composite quality indexes and assumed costs under Scenario A, while Table 7 depicts the same information under Scenario B. The quality indexes are computed using the unweighted and DEA-based weighting based on the consensus scores formulated by the hypothetical expert panel as well as individual scores from the six experts. We find that DEA has low discriminatory power, failing to distinguish between DCMs. This is mostly due to the low number of observations. The individual scores from the six experts help overcome this challenge by multiplying the number of observations by six, which improves the discriminatory power of the quality index and improving its overall reliability.

Under Scenario A, both CEA and EA produced consistent rankings of the DCMs under evaluation (see Table 8). HMS is ranked as the most cost-effective/cost-efficient system, followed by census. Surveillance-type systems usually obtain middle rankings, and surveys like DHS and NHS are consistently the least cost-effective methods to gather vital statistics. Even though the results were based on simulated data, they seem plausible, particularly given that we used the number of vital events recorded as the measure of quantity of data to be produced. Given that this choice of quantity was justified based on the long-term government policy of establishing CRVS, it is not surprising that HMS is relatively more cost effective/cost efficient than individual surveys that rely on samples of the population, with sometimes low coverage and/or limited small-area statistics.

**Table 8:** Results for Scenario A.

DCM	Cost Effectiveness Analysis						Efficiency analysis		
	Cost per QADI:			Cost per QADI:			Cost-efficiency index		
	Unweighted quality index			DEA-based quality index					
	Consensus matrix	Individual matrix	Rank	Consensus matrix	Individual matrix	Rank	Consensus matrix	Individual matrix	Rank
Census	0.0847	0.0827	2	0.0644	0.0644	2	1.0000	0.9667	2
NHS	3.5117	3.3812	8	2.0610	2.0649	8	0.0280	0.0248	8
DHS	8.4740	8.0615	9	5.8000	5.8000	9	0.0100	0.0102	9
HMS	0.0313	0.0295	1	0.0259	0.0259	1	1.0000	1.0000	1
IDS	0.2943	0.3152	5	0.2250	0.2302	5	0.1630	0.1585	5
X-DSS	1.2925	1.3288	7	1.2121	1.2172	7	0.0490	0.0485	7
Y-DSS	1.0602	1.0602	6	1.0602	1.0602	6	0.0570	0.0563	6
MM	0.1385	0.1370	3	0.1200	0.1208	3	0.4040	0.3817	3
A-DSS	0.2190	0.2046	4	0.1957	0.1957	4	0.2360	0.2410	4

*Notes*: See Table 4 for definitions and further details on the DCMs. DMC, data collection method; QADI, quality-adjusted data index; DEA, Data Envelopment Analysis.

Results for Scenario B are presented in Table 9. The relative rankings of alternative DCMs by CEA and EA are consistent overall. For example, both methods ranked NHS as the most cost-effective/best performing system, followed by the nationally representative sample, DHS. On the other hand, DSSs were identified as the least cost-effective method to gather vital statistics, which is not surprising given their low coverage. Interestingly, CRVS, as a method of collecting vital statistics is relatively cost efficient, particularly as it incorporates COD reporting.

**Table 9:** Results for Scenario B.

DCMs	Cost Effectiveness Analysis						Efficiency analysis		
	Cost per QADI:			Cost per QADI:			Cost efficiency index		
	unweighted quality index			DEA-based quality index					
	Consensus matrix	Individual matrix	Rank	Consensus matrix	Individual matrix	Rank	Consensus matrix	Individual matrix	Rank
Census	0.0802	0.0828	6	0.0644	0.0644	6	0.0630	0.0580	5
NHS	0.0022	0.0023	1	0.0015	0.0015	1	1.0000	1.0000	1
DHS	0.0034	0.0036	2	0.0026	0.0026	2	0.8050	0.9113	2
CRVS	0.0312	0.0323	5	0.0145	0.0136	4	0.1930	0.1738	6
HMIS	0.0177	0.0154	3	0.0142	0.0135	3	0.5540	0.4513	3
IDS	0.1514	0.1574	7	0.1125	0.1154	7	0.0430	0.0320	7
X-DSS	0.1682	0.1690	8	0.1601	0.1571	8	0.0490	0.0353	8
Y-DSS	0.4835	0.4835	10	0.4835	0.4835	10	0.0190	0.0128	10
MM	0.0266	0.0266	4	0.0231	0.0234	5	0.2940	0.2213	4
A-DSS	0.2094	0.2030	9	0.1957	0.1957	9	0.0390	0.0307	9

*Notes*: See Table 4 for definitions and further details on the DCMs. DMC, data collection method; QADI, quality-adjusted data index; DEA, Data Envelopment Analysis.

## Discussion

This study presents the first documented framework to determine the most efficient method to collect vital statistics data. While the literature does provide a framework to assess the quality of vital statistics data [2], this framework still needs to be operationalised so that it can be used to measure the quality of data of available DCMs. Moreover, no previous study has attempted to systematically examine the costs of collecting vital statistics data. We identified two major economic approaches – cost effectiveness analysis and efficiency analysis – to systematically assess DCMs and outlined the necessary context assessment required to utilise these approaches.

Using simulated data, the results of the two stylised scenarios showed that the rankings of the DCMs are not affected by the choice of economic approach. However, a comparison of the results from Scenarios A and B did show very different rankings of DCMs. For example, surveys are found to be the most cost-efficient methods in Scenario B, yet they are the least efficient in Scenario A. This is driven by the choice of measure of ‘quantity’ of vital statistics data. In Scenario B, we use target population or number of persons represented, not number of unit records as used in Scenario A. Such a measure favours those methods with large coverage and relatively smaller samples, such as nationally representative surveys. They would be measured as producing large output (as measured by coverage) with relatively low input (sampled population). This is, however, in line with our assumptions of national policy focused on either the long-term establishment of CRVS (Scenario A) or on collecting vital statistics data representative at national level (Scenario B).

Several caveats concerning this exercise should be noted. First, this exercise is a snapshot evaluation of DCMs. It answers the question ‘Which DCMs are the most cost-effective/cost-efficient for vital statistics data in a particular (hypothetical) setting? ’ As explained in the File S2, our evaluation does not answer the question ‘Is the goal of collecting vital statistics optimal given other social or development objectives? ’. Such a question would involve the highly contentious task of assigning monetary values to the benefits of funding vital statistics. Similarly we do not answer the question of ‘Which method will be the most cost-effective/cost-efficient to achieve universal coverage of vital statistics data? ’ Answering this would require a good understanding of the marginal costs and the cost functions of the various DCMs, which are largely unknown. On the other hand, we should stress that the first step is to understand the current cost structure of alternative DCMs in light of their outputs. This paper proposes a systematic and rigorous way to undertake such analysis, which can be tested in-country.

Second, any systematic evaluation of the outputs of DCMs would need to capture the quality of the data produced. However, quality does not have an intrinsic measurement scale and its quantification is highly subjective. It is thus important to be as transparent as possible and acknowledge that the evaluation will always have an important element of subjectivity.

Third, there is no perfect measure of the quantity of output produced by DCMs and the choice of measure can have a substantial influence on the results and the corresponding rankings. In our case, we have proposed two different measures of quantity of data and provided a clear policy rationale for choosing one over the other in particular settings.

There have been several calls for global improvements in collecting useable data from health information systems to inform public health policies. Chief among these is the need to improve current standards of information on vital statistics and COD. As yet there is no framework to help countries undertake an economic evaluation of DCMs and establish which alternative provides the best value for money. Indeed, this topic seems under-researched given the large investments made by donors and governments to various DCMs and health information systems in general. This might be partly explained by the numerous challenges discussed throughout the paper. These range from problems of methodology, such as the definition of quality, to implementation factors such as the lack of basic costing data.

To the best of our knowledge, ours is the first study that provides a systematic framework to compare outputs and costs of alternative DCMs. This systematic assessment of the elements required a rigorous economic evaluation of DCMs and is also the first in the area of health information systems. Our study examined the elements and challenges of the proposed framework, while also providing some feasible approaches to deal with the challenges. In doing so, we laid out the assumptions in a transparent manner and built a stylised scenario of a hypothetical low-income country to illustrate how the proposed framework might operate in country applications. As such, our study provides information on the basic parameters and modelling assumptions required to build simulation models to aid decision-making on future investments in collecting vital statistics data. Similar types of simulation models, such as LiST or the Investment Case Matrix, have been used to guide decision-making in other areas of health [15], [16]. Our work is still in its infancy and much remains to be done, particularly to pilot test the proposed framework in an individual country using actual, rather than simulated, data. However our exercise demonstrates that a systematic evaluation of outputs and costs of DCMs is not only necessary, but also feasible.

At a time when the global development community is demanding sound evidence to inform its investments, the lack of information on the relative costs and benefits of investing in vital statistics is hindering global efforts to strengthen CRVS. Our study and the proposed framework are a first, vital step to fill this gap and should prompt country applications that inform decision-making on how much and where to invest resources for vital statistics. These country applications of our framework can be used to engage stakeholders in evaluating the alternatives available and developing investment cases for vital statistics. This will provide a focus around which to rally the currently scattered, but increasingly serious efforts to strengthen birth, death and cause of death systems worldwide, and guide the critical investment decisions of the global donor community.

## Supporting Information

File S1 **Supporting Information file containing: Appendix S1, Detailed overview of the framework for assessing costs and outcomes of DCMs.**
(DOCX)

File S2 **Supporting Information file containing: Appendix S2, Technical overview of the quantitative methods. Figure S1, Frontier constructed using DEA technique: efficient vs. inefficient DMUs. Box S1, Estimation Process.**
(DOCX)

File S3 **Supporting Information file containing: Dataset S3, computer-generated data constructed for the simulated examples.**
(XLSX)
