# Supplementary material for: A Framework for the Economic Analysis of Data Collection Methods for Vital Statistics
Source: PLoS One. 2014 Aug 29;9(8):e106234. doi: 10.1371/journal.pone.0106234 (PMC4149535; doi:10.1371/journal.pone.0106234)
Supplement: File S1 — Supporting Information file containing: Appendix S1, Detailed overview of the framework for assessing costs and outcomes of DCMs. (DOCX) [file pone.0106234.s001.docx]

**FILE S1: SUPPORTING INFORMATION**

**Appendix S1: Detailed overview of the framework for assessing costs and outcomes of DCMs**

In this supplementary file, we provide details on the methodology utilised in this study. As noted in the main text, four elements are involved in the proposed framework grouped into two major areas. The ‘context assessment’ encompasses the first three elements and involves the collating of the necessary information to perform the economic analysis. The ‘quantitative assessment’ covers the fourth element and consists of a systematic appraisal of the comparative costs and outcomes/benefits of the various methods using two economic approaches. We outline each element in detail below. Further details on the quantitative methods are presented in File S2.

**Element 1: Identify the alternatives to be evaluated**

The first element of the proposed framework consists of a literature search to identify the various alternatives to be evaluated, which in our case are the methods to collect data on vital events. We searched the MEDLINE and SCOPUS databases using a combination of the MESH terms ‘vital statistics’ and ‘data collection’. We then examined the resulting 256 references published since 1970 for relevance. We checked the reference lists of selected articles to ensure that we had included all relevant papers. We identified three major categories of DCMs for obtaining mortality information when complete CRVS is not available. The first category was termed ‘partial registration’, which involved the registration of births and deaths within a subset of the population. Two types of partial registrations were uncovered: (a) Sample Registration System (SRS), which entailed covering a nationally representative subset of the population by a complete registration system within particular sites [[1](#_ENREF_1),[2](#_ENREF_2)]. India’s SRS system [[3](#_ENREF_3)] and China’s Disease Surveillance Points (DSP) system [[4](#_ENREF_4)] are examples of this method. (b) Demographic Surveillance Sites (DSS), which are registrations set up in settings where even an SRS has been too difficult to implement and are representative only of a major typology [[5](#_ENREF_5)].

‘Censuses and population surveys’ were the second major source of mortality data observed. Censuses are conducted every 10 years and provide an almost complete record of the total population at the time of the survey [[6](#_ENREF_6)]. Population surveys, such as the Demographic and Health Survey (DHS), are conducted more frequently (approximately every five years) but survey only a representative sample of the population. Depending on the questions asked in the survey, mortality rates can be determined either directly or indirectly [[7-9](#_ENREF_7)].

The final major category was ‘facility-based data collection’. These sources relied on recording deaths that occur in health facilities [[10](#_ENREF_10)]. These sources of data are heavily dependent on the proportion of the population that use the facilities and are not usually accurate sources for mortality rates, unless the coverage of health services is high [[11](#_ENREF_11)]. Further details on sub-categories are provided in Table 1 of the main text.

**Element 2: Identify and measure the outcomes of the alternatives to be evaluated**

One of the basic elements of a sound economic evaluation is the choice of appropriate physical units to measure outcomes [[12](#_ENREF_12)]. In general, the aim of DCMs is to produce good quality data for estimating vital statistics. Therefore, any proposed measurement for the outcomes of DCMs would need to incorporate, in addition to quality, the quantity dimensions of the data.

*Measuring outcomes: quantity*

In some cases identifying the ‘quantity of data’ will be relatively straightforward. For example, mortality can be measured as number of deaths. Unfortunately, there is no clear guidance regarding the appropriate units to measure the quantity outcomes produced by DCMs. For example, should one use the number of vital events that get registered as units of measurement? This seems reasonable when quantifying the outcomes of the CRVS. Or should one count the number of people represented in the statistics produced by each method? This might seem reasonable for household surveys.

We propose that the choice of units of measurement is informed by the government policy that relates to CRVS. Where the long-term policy is to establish CRVS, we propose that ‘quantity of data’ is defined as ‘the unit records^^[[1]](#footnote-1)^^ collected by each DCM’. Such a metric would be in line with the long-term policy of CRVS, which would seek to collect all unit records of vital statistics for a country’s population. In this case, the number of unit records could be approximated by the sample size of the DCM.

On the other hand, where CRVS is not part of the government’s long-term policy, it seems reasonable to assume that the government does not place high value on obtaining unit records for all its citizens and so a different metric should be considered. In this event, a metric such as ‘the number of people represented by each DCM’ might be more suitable and in line with the need to produce vital statistics that are representative of the population, and that can be easily obtained from the documentation commonly provided with each DCM.^^[[2]](#footnote-2)^^

*Measuring outcomes: quality*

A systematic assessment of quality should be two-fold. First, to clearly identify those attributes that define the quality of the alternatives under consideration, and second to operationalise those attributes so that they can be measured for each alternative. That is, for each attribute we need to define clear evaluation criteria and provide an adequate measurement scale.

Our assessment of the quality of each DCM is built on the assessment framework of Mahapatra *et al.* [[13](#_ENREF_13)], which built on the framework proposed by Ruzicka and Lopez for evaluating quality of mortality and COD data [[6](#_ENREF_6)]. The framework has five quality attributes: (1) accuracy, determined by issues such as coverage, completeness, missing data, use of ill-defined or improbable categories and consistency with other estimates; (2) relevance, measured by the applicability of the data to age, sex and geographical groupings; (3) comparability, encompassing standard coding practices and definitions; (4) timeliness, measured by the production time and regularity of data releases; and (5) accessibility, the ease of access to data. We split each of these five assessment categories into criteria that assess the collection of statistics related to vital events (i.e. births and deaths) and the collection of statistics related to COD (see Table 2 of the main text).

The quality attributes proposed by Mahapatra *et al.* [[13](#_ENREF_13)] are in line with those included in quality frameworks for official statistics. Thus, although originally developed for CRVS, it seems reasonable to expect that criteria based on this framework can be applied to other methods of collecting vital statistics. However, we need to address two important limitations of this framework in relation to our study.

First, as discussed previously, the scope of the evaluation is defined by the government’s policy on CRVS. If the long-term policy is the establishment of complete CRVS, then the criteria used to evaluate the interim methods used to collect data on vital statistics should include the extent to which they enable or undermine such a policy. Enabling or undermining the long-term policy of setting up CRVS is not an attribute of the quality of the data produced and so is not included in the Mahapatra *et al.* [[13](#_ENREF_13)] framework. It is an effect, however, and a very important one, of alternative methods of collecting vital statistics data. Therefore, we suggest that ‘improvement towards CRVS’ is added as one of the criteria used to evaluate the ‘quality’ of the outcomes of DCM, but only if such a policy is in place.

Second, to provide a numeric measure of quality, we need to establish an appropriate ordinal scale of the potential scores that can be assigned under each criterion. This would allow us to assess each DCM under each quality criterion and provide comparative scores to rank the different DCMs. Such a range would be indicative only. It is important to ensure that the range of scores and the corresponding cut-offs assigned differentiate the degrees of quality. In country applications, it might be advisable for the quality scoring matrix to be developed by expert consensus. Another option is developing global standard quality scoring matrices for various typologies of countries. This would facilitate cross-country comparisons and save time and resources.

To implement a straightforward approach and to facilitate discussion, we assumed a basic linear scoring system with a range of [0–10] applied to each quality criteria. For instance, we defined the score range for coverage, as 0 = 0% (or indicators not collected), 1 = 0+ to 10%, 2 = 10+ to 20%, …, 10 = 90+ to 100%. So, if a particular DCM in our hypothetical country covers 5 million people, then its coverage rate would be 15 per cent and therefore the score would be 2. Arguably, a finer scoring system is possible and would provide more variation in the scores of alternative DCMs. However, given the reliance on expert opinion for the quality assessment, these finer score ranges might be deceiving. On the other hand, very broad score ranges may not give enough variation in the quality scores of alternative DCM and so might not be very informative. Given the potential subjectivity of assigning quality scores to each DCM, we suggest that in-country evaluations use a team of experts to assign the scores either by consensus (i.e. using Delphi techniques) or individually (i.e. aggregating the individual scores from experts into a single score).

As outlined in the main text, the quality scores assigned to each DCM in the stylised examples are hypothetical, based on assumptions of what might be plausible in a hypothetical low-income country. We provide a brief description of these assumptions below.

For our hypothetical country, DCMs did not score highly for ‘accuracy’, partly because we assumed their coverage to be low (see Tables 6 and 7 in the main text). Additionally, in trying to reflect the situation in many countries, we assumed that most DCMs, except for data surveillance systems (e.g. X-DSS, Y-DSS and MM), did not collect information about COD, or did not have recorded deaths with appropriately certified COD (incomplete) or most death records had ill-defined COD (use of ill-defined categories). However, the consistency between COD and general mortality of most DCMs was assumed to be fairly high with estimates expected to be within two standard deviations of general mortality-based predictions. Likewise, the scores given against ‘relevance’ were not very high, as we assumed that only a few DCMs collected and produced statistics by gender and standard age groups. We assumed surveillance-type systems (e.g. X-DSS, Y-DSS and MM) were representative at the lowest administrative level within the corresponding surveillance sites, thus receiving relatively higher scores.

Similar to the situation in many low-income countries, the definitions and estimations of mortality and COD indicators changed gradually over time and varied across the surveillance areas. Comparability across space was also assumed to be relatively low, as it might be influenced by the capacity of the staff working on reporting and coding death events. Therefore, most DCMs are given a ‘consistency’ score between 3 and 6. In assessing the ‘timeliness’ of the various DCMs, the systems that are typically designed to collect data continuously and produce statistics within a year interval were given the high scores of 9 or 10. Moreover, most DCMs provide datasets in various formats and with average quality documentation of the data collection process, due to incomplete documentation. The user services are not always available and timely. Therefore, the overall score given to ‘accessibility’ ranges between 4 and 7. Lastly, facility-based systems, such as health management systems and integrated disease surveillance, are given relatively high scores for ‘improvements toward a complete CRVS’. By contrast, the DHS and National Household Surveys were assigned low scores as their data collection designs and objectives (e.g. income and poverty) are further removed from the aims of CRVS.

**Element 3: Identify and measure the costs of the alternatives to be evaluated**

The third element of the proposed framework is to assess the comparative costs of using the alternatives. However, with respect to DCMs, these costs are not well documented. There is a vast amount of literature on methodologies for costing exercises, particularly in health services [[12](#_ENREF_12)]. In fact, a major hindrance for development partners and governments trying to make informed decisions on investments in DCM is the absence of costing data and appropriate costing frameworks for DCM [[14](#_ENREF_14)]. Given how little we know about the costs associated with DCMs, an important first step would be for countries to start measuring the cost of their systems.

The two basic approaches for measuring costs are bottom up – whereby the resource items are identified and information on the associated quantities and unit prices is used to obtain the total cost of collecting vital statistics data – and top down – which relies on first estimating the total costs of a system, usually from budget documents (i.e. the total cost of operating the CRVS) and then using appropriate allocation rules to allocate total costs to individual functions (i.e. which proportion of the CRVS costs should be allocated to producing vital statistics). Bottom-up costing is usually the preferred method, since it can produce relatively accurate estimates [[15](#_ENREF_15),[16](#_ENREF_16)]. However, it is not always feasible. A recent exercise in South Africa illustrates the practical and methodological challenges in capturing even basic cost elements of CRVS [[14](#_ENREF_14)]. The report’s authors found that government estimates of direct costs involved in registering a birth were available for staff time and consumables, but not for indirect costs and overheads, which were likely to be substantial. However, for top-down costing the allocation or apportioning rules chosen will have a significant impact on the costing estimates. They should thus be transparent, documented in detail and follow a clear logic that is easy to justify. Most importantly, the rules should consider the various outputs produced by each DCM and provide a clear justification as to why a relatively large or small proportion of costs should be allocated to the production of vital statistics. Indeed, most DCMs produce many outputs, only one of them being vital statistics. Therefore a combination of bottom-up and top-down costing would most likely be required.

The evidence on costing of DCMs is so limited that little guidance exists beyond the general costing textbooks and manuals [[15](#_ENREF_15),[16](#_ENREF_16)]. Additionally, estimating costs when limited or aggregated data only are available usually involves a considerable number of assumptions and subjective modelling decisions. Therefore, in order to facilitate the replicability of the results and the rigour of the costing exercise, it is important to be as systematic as possible and have detailed documentation of the assumptions used and their justification. Understanding the costing structure of each DCM (i.e. how much it is costing to collect the observed amounts and quality of vital statistics data) is an important first step. However, we are also interested in the cost functions of each DCM (i.e. how costs change with changes in output, output being the number of events being recorded or coverage).

Although we know little about the cost functions of the DCMs, there are a few reasonable assumptions that we can make. First, it is likely that those costs would be non-linear and discontinuous. For example, it is reasonable to expect that the costs of CRVS will not increase in a constant proportion to the number of events recorded (i.e. the costs of recording 80% of the population vital events will not be eight times the costs of covering 10%). We can also expect that there will be thresholds/points at which large additional fixed costs are required (i.e. once a certain level of coverage is achieved, significant investments in information technology and a dedicated team of analysts may be required). Second, new technologies are likely to have substantive impacts on the expected costs and feasibility of DCMs, such as CRVS, which are difficult to predict. This is illustrated by the example of the highly ambitious ‘Unique Identification’ scheme launched in India in 2010, which could in the future be linked to CRVS. Notwithstanding the challenges, this scheme seems to be paving the way for the creation of unduplicated identity numbers linked to basic biometric and demographic data at a reported cost per person enrolled of US$2 [[17](#_ENREF_17)]. Third, from what we know about DCMs, the relationship between costs and output will also likely vary from one DCM to the next. For example, for a survey it is reasonable to assume that the population sample might be the main cost driver, but this is unlikely to be the case for sample registration, where the number of sample units might be the main cost driver. Fourth, cost functions will likely be highly context specific and so will vary significantly between countries. Fifth, in some instances where there are regional variations in coverage (e.g. facility-based reporting covers 80% of the population in a particular province, but only 30% and 10% in others), this information might be used to approximate the cost function of a particular DCM in that country. In this event, it will be possible to predict how costs will change as coverage/number of events recorded increase in that particular setting. However, in many cases this information will not be available.

Since we do not have information on the parameters of the cost functions of alternative DCMs, we will only be able to estimate the costs associated with current coverage. This means, we will be able to answer the question of ‘which DCM is currently providing the best vital statistics data given the associated costs’, but not the question of ‘which DCM will be the most efficient to achieve *x* number of records or *y* per cent of coverage’. However, if countries understand the current costing structure of their alternative DCMs, this information can be used to examine, at least in hypothetical scenarios, what the most efficient DCM will be to achieve the targeted coverage. To do so, the parameters of the current costing structure can be used to build basic simulation models that also rely on specific assumptions about threshold costs. Other parameters can be used to predict the future costs of scaling-up alternative DCMs. This type of simulation has been used in other areas of policymaking, including health, where simulation models aid decision-making [[18](#_ENREF_18),[19](#_ENREF_19)]. However, even the most basic simulation model would require information on the current costing structure of each DCM.

Lastly, each DCM has different objectives and outputs, so it is important to allocate the share of total costs to the production of vital statistics data. This is a challenging task for several reasons. First, there is a large degree of uncertainty about the cost of vital statistics collection as a proportion of the total cost of each DCM. We decided to approximate this using the share of vital statistics indicators produced. For instance, of the 13 types of indicators that the HMS collected, two of them relate to vital statistics. Therefore the resource share is approximately 15 per cent. However, collecting data on some types of indicators might require more resources than others. For instance, collecting vital statistics via verbal autopsy instruments tends to be more resource intensive than collecting data on use of the health service. In such cases, the collection of data on two vital indicators (out of 13) might take a resource share higher than the above mentioned 15 per cent, and the apportion rule should be adjusted upwards to reflect this. Second, in reality there might be different rules to apportion different types of costs (i.e. fixed vs. variable).

**Element 4:** **An economic assessment of the alternatives to be evaluated**

The final element of the framework consists of two economic tools which utilise the information collated in elements 1 to 3 to quantitatively assess the comparative effectiveness and efficiency of the various alternatives.

The first economic tool employed is cost-effectiveness analysis (CEA), which as detailed in the File S2, is one of the most commonly used economic evaluation methods in health [[12](#_ENREF_12)]. CEA aims to assess both the costs and outcomes of competing alternatives without relying on monetary units to measure the latter [[12](#_ENREF_12),[20](#_ENREF_20)]. However, since the alternatives (i.e. health interventions) being evaluated have very different health benefits/outcomes, one must develop or rely on indicators, such as quality-adjusted life-years (QALYs), which standardise the outcomes to be evaluated and capture the health benefits. We are in a similar situation when evaluating alternative DCMs. These methods produce different types of vital statistics data of varying quality, according to the different criteria as outlined above. Since we have multiple criteria, we could reasonably expect that some methods are superior to others when ranked by a particular quality criterion, but not by others. Therefore, to enable the systematic comparison of DCMs outcomes via CEA, one would need to aggregate the scores into a single index of quality that could then be used as a standardised metric of the DCMs outcomes. We should note that in evaluating health interventions, the number of deaths averted – without adjustments for the quality of life – is sometimes used. However, in our case, an index that does not capture quality would be quite problematic since it would imply that large amounts of data of very poor quality – and consequently, of limited use – are preferable to smaller amounts of data of very high quality. On the other hand, an index that does not capture quantity would fail to provide us with appropriate physical units to measure outcomes. The latter would be equivalent to trying to quantify the consequences of health interventions by using quality of life, but without measuring the number of years of life gained.

We have identified three alternative methods that can be used to produce a composite index: (1) an unweighted average; (2) a weighted average, where weights are identified *a priori* by experts; (3) a Data Envelopment Analysis (DEA) based index. Given that no method is superior, we test the robustness of our results comparing the rankings formed using a composite index constructed using DEA versus one built from the unweighted mean scores [[21](#_ENREF_21)]. Note that in this exercise, we did not have a team of experts to assign weights to each criterion. We strongly recommend using expert opinion to weight indexes when possible, and that the process of assigning weights is kept explicit and transparent. To combine the quantity and quality dimensions, the quality index score is multiplied by the quantity indicator. This provides the quality-adjusted data index (QADI) produced by each DCM. When combined with the cost data, we can produce the ‘cost per QADI’ for each DCM. This ratio can be used to rank the alternative DCMs under evaluation.

The second economic tool employed is efficiency analysis. In general, this analysis uses econometric techniques to estimate and compare the productivity and efficiency of outputs from a given set of inputs [[21](#_ENREF_21)]. We frame our evaluation problem as follows: (a) Each DCM can be considered a unit of production, (b) DCMs produce the outputs of ‘good quality vital events data’, in which the ‘good quality’ of data can be measured by different quality attributes and the output is data, (c) The inputs used by each DCM are defined as the resources required to collect the relevant data. For example, human resources, facilities and equipment, and those inputs can be measured in monetary terms and so can be aggregated into a single item of input, which is the total cost associated with each DCM, (d) We use outputs (quantity and quality of data produced) and inputs (costs) to evaluate the productivity of each DCM; that is, how much ‘good data’ each DCM can produce given the associated cost. Each DCM has a maximum productivity; that is, the highest possible quality data it can achieve given the amount of resources. Therefore, we can rank DCMs from best (most productive) to worst (least productive). In other words, we are interested in the relative rank of DCMs in terms of their productivity or cost efficiency.

In contrast to CEA, an analyst using efficiency analysis techniques does not need to separately build a composite quality index. EA can handle multiple outputs by using the in-built assumptions in DEA to combine information on multiple outputs and costs. DEA uses linear programming to estimate a cost-efficiency index that combines outputs (i.e. quality scores against each attribute and quantity of data) and the associated costs of each DCM. This cost-efficiency index is then used to produce the corresponding ranking of DCMs. The interpretation of this index is straightforward; the range of this index is [0–1] and the closer the index to unity, the more cost efficient the DCM. So we rank the data collections by how close they are to unity. Overall, this systematic assessment provides a rigorous economic evaluation of DCMs and is the first of its kind in the area of health information systems.

**References**

1. Mozumder KA, Koenig MA, Phillips JF, Murad S (1990) The Sample Registration System: an innovative system for monitoring demographic dynamics. Asia-Pacific Population Journal 5: 63-72.

2. Setel PW, Sankoh O, Rao C, Velkoff VA, Mathers C, et al. (2005) Sample registration of vital events with verbal autopsy: a renewed commitment to measuring and monitoring vital statistics. Bulletin of the World Health Organization 83: 611-617.

3. Mari Bhat PN (2002) Completeness of India's sample registration system: an assessment using the general growth balance method. Population Studies 56: 119-134.

4. Rao C, Lopez AD, Yang G, Begg S, Ma J (2005) Evaluating national cause-of-death statistics: principles and application to the case of China. Bulletin of the World Health Organization 83: 618-625.

5. Pena R, Perez W, Melendez M, Kallestal C, Persson L-A (2008) The Nicaraguan Health and Demographic Surveillance Site, HDSS-Leon: a platform for public health research. Scandinavian Journal of Public Health 36: 318-325.

6. Ruzicka LT, Lopez AD (1990) The use of cause-of-death statistics for health situation assessment: national and international experiences. World Health Statistics Quarterly - Rapport Trimestriel de Statistiques Sanitaires Mondiales 43: 249-258.

7. Rajaratnam JK, Tran LN, Lopez AD, Murray CJL (2010) Measuring under-five mortality: validation of new low-cost methods. PLoS Medicine 7: e1000253.

8. Hill K, El Arifeen S, Koenig M, Al-Sabir A, Jamil K, et al. (2006) How should we measure maternal mortality in the developing world? A comparison of household deaths and sibling history approaches. Bulletin of the World Health Organization 84: 173-180.

9. Obermeyer Z, Rajaratnam JK, Park CH, Gakidou E, Hogan MC, et al. (2010) Measuring adult mortality using sibling survival: a new analytical method and new results for 44 countries, 1974-2006. PLoS Medicine 7: e1000260.

10. Hill K, Lopez AD, Shibuya K, Jha P (2007) Interim measures for meeting needs for health sector data: births, deaths, and causes of death. The Lancet 370: 1726-1735.

11. Boerma JT, Stansfield SK (2007) Health statistics now: are we making the right investments? The Lancet 369: 779-786.

12. Drummond M, Sculpher M, Torrance G, O'Brien B, Stoddart G (2005) Methods for the economic evaluation of health care programmes. USA: Oxford University Press. 379 p.

13. Mahapatra P, Shibuya K, Lopez AD, Coullare F, Notzon FC, et al. (2007) Civil registration systems and vital statistics: successes and missed opportunities. The Lancet 370: 1653-1663.

14. Cambridge Economic Policy Associates (2013) Health Metrics Network: Estimating the Cost of the Civil Registration and Vital Statistics System in South Africa. London: Cambridge Economic Policy Associates.

15. Mogyorosy Z, Smith P (2005) The main methodological issues in costing health care services: A literature review. York: CHE Research Papers No. 7, University of York.

16. Rommelmann V, Setel PW, Hemed Y, Angeles G, Mponezya H, et al. (2005) Cost and results of information systems for health and poverty indicators in the United Republic of Tanzania. Bulletin of the World Health Organization 83: 569-577.

17. The Economist (2012) The magic number: India’s identity scheme. The Economist. United Kingdom: The Economist Newspaper Ltd.

18. Jimenez Soto E, La Vincente S, Clark A, Firth S, Morgan A, et al. (2012) Developing and costing local strategies to improve maternal and child health: the investment case framework. PLoS Medicine 9: e1001282.

19. Winfrey W, McKinnon R, Stover J (2011) Methods used in the Lives Saved Tool (LiST). BMC Public Health 11 Suppl 3: S32.

20. Muennig P (2008) Cost effectiveness analysis in health: A practical approach. San Francisco: Jossey-Bass. 266 p.

21. Coelli TJ, Rao DSP, O'Donnell CJ, Battese GE (2005) An introduction to efficiency and productivity analysis. New York: Springer.

1. Each record represents an event (often the case for administrative data) or a person’s specific details (often the case for population data). [↑](#footnote-ref-1)
2. We should note that although small-area estimates are not accounted for by the size of the population represented by the data, they are accounted for in the quality index proposed. [↑](#footnote-ref-2)
