# Supplementary material for: A Framework for the Economic Analysis of Data Collection Methods for Vital Statistics
Source: PLoS One. 2014 Aug 29;9(8):e106234. doi: 10.1371/journal.pone.0106234 (PMC4149535; doi:10.1371/journal.pone.0106234)
Supplement: File S2 — Supporting Information file containing: Appendix S2, Technical overview of the quantitative methods. Figure S1, Frontier constructed using DEA technique: efficient vs. inefficient DMUs. Box S1, Estimation Process. (DOCX) [file pone.0106234.s002.docx]

**FILE S2: SUPPORTING INFORMATION**

**Appendix S2: Technical overview of the quantitative methods**

In this supplementary file, we provide a brief technical overview of the quantitative methods employed to assess the comparative effectiveness and efficiency of the various data collection methods: cost-effectiveness analysis (CEA) and efficiency analysis, namely Data Envelopment Analysis (DEA). Here we focus on outlining the details of the methodology which are relevant for the purposes of undertaking an economic evaluation of data collection methods (DCMs).

###

### Economic evaluation: cost-effectiveness analysis

Economic evaluation is concerned with the systematic and comparative assessment of costs and benefits/outcomes of alternative options. There are three major methods available: cost-benefit analysis; cost minimisation analysis; and cost-effectiveness analysis [1]. They are briefly summarised below:

Cost-Benefit analysis (CBA) is a method used to measure and compare the social benefits and costs of a particular project or program [1,2]. CBA requires that all costs and consequences are expressed in the same monetary units. Although widely applied and used by governments around the world, CBA is not directly applicable to the evaluation of DCMs. CBA would require the analyst to assign a dollar value to the benefits of obtaining vital statistics data. In our case, this is clearly unfeasible as it would require a measurement of the impact that good quality statistics of vital events have on decision-making (i.e. was the data used to inform specific decision-making and if yes, what was their contribution?) and health outcomes (i.e. what proportion of the improvements in health can be attributed to those evidence-based decisions). Additionally, it could be argued that in the case of vital statistics data, there is social consensus in regard to their added social value, which does not need to be established via economic evaluation [3,4]. In this case, the use of CBA becomes redundant.

Cost minimisation analysis aims to identify the least costly alternative to achieving the same outcome [1,5]. It requires that the alternatives under assessment have the same outcomes (e.g. apply the same questionnaire on vital statistics to 20,000 households) and only differ by inputs/costs (i.e. interviewers filling out paper questionnaires vs. interviewers entering data into their computers). Cost minimisation analysis is not applicable in our context since various DCMs have different outcomes (e.g. surveys would produce vital statistics representative at national level, but not at district level, while the opposite might be true for a disease surveillance system).

Similar to CBA, cost effectiveness analysis (CEA) aims to assess both the costs and outcomes of competing alternatives, but does not rely on monetary units to measure the latter [1,5].[[1]](#footnote-1) CEA is used when one’s objective is to compare options with similar outcomes/outputs. In many instances, the health interventions being evaluated have very different health benefits/outcomes (e.g. heart surgery vs. a diabetes treatment). The analyst would then need to develop or rely on indicators, which standardise the outcomes to be evaluated and allow the capture of the different health benefits. In our context, such index, similar to QALYs, needs to capture both the quality and the quantity aspects of the vital statistics produced by each DCM. Details on the quality-adjusted data index (QADI) developed to quantify the output of each DCM is provided in File S1. Using this index and corresponding cost data, we compute ‘the cost per quality-adjusted data index’ for each DCM and this ratio is used to rank the alternative DCMs.

###

### Efficiency Analysis: Data Envelopment Analysis (DEA)

Another relevant literature for our research problem is the benchmarking and efficiency analysis literature [6]. It looks at measuring outputs, inputs and the productivity of production units (e.g., firms, hospitals, schools, government offices), and uses econometric techniques to estimate and compare the productivity and efficiency of those units. The literature is built on a number of basic concepts from production economics, including the production frontier and total factor productivity. Efficiency in this literature is defined as the ability to achieve maximal outputs from a given set of inputs [6,7].

There are many different methods available in the literature to estimate efficiency. Data Envelopment Analysis (DEA) is one popular method, particularly suitable for our purposes because it is a data-oriented approach that produces implicit weights for each decision making units (DMU) – i.e. each data collection method in our framework. Consequently, one does not need to rely on *a priori* preferences on the relative importance of different quality criteria. Unlike other methodologies, DEA requires very few assumptions, which facilitates its use in cases involving complex (and often unknown) relations between multiple inputs and multiple outputs.

Let’s now formally outline the model for the case of a set of $N$DMUs, each using one input to produce $M$outputs. We can define the output distance function as follows:

where $q_{i,m}$ is the amount of output $m$produced by the DMU $i$; $\lambda_{i}$ is the weighting factor; $x_{i}$ is the amount of input used;[[2]](#footnote-2) and the restriction of $\sum_{i} \lambda_{i}=1$allows for variable returns to scale. The scalar $\varphi$ represents by how much the production each DMU can increase its output by holding input constant; that is the proportional increase in output that could be achieved by DMU $i$ without increasing its input consumption. The ratio of $\frac{1}{\varphi}$ defines the technical efficiency score and it takes the value between 0 and 1. If $x_{i}$ is the total cost, the score is equivalent to a cost efficiency score.

The efficiency score is illustrated graphically in Figure S1. Four DMUs produce two outputs Q1 and Q2 using the same amount of one input (X or total cost). Three DMUs – A, B and C – form the production frontier and D sits below the frontier (i.e. inside the production possibilities set). This implies that A, B, and C are fully efficient, and the main difference in their production processes is the mix of outputs that they produce. For example, unit A chooses to produce more Q1 than unit C, while its production of Q2 is around a quarter of that of unit C. However, we can say that “given their choice of output mix, they are doing the best they can”. On the other hand, D is not fully efficient, i.e. it uses the same amount of input as A, B, and C but produces substantially less of both Q1 and Q2.[[3]](#footnote-3)

When applying DEA for this data, it will result in A, B, and C having the efficiency index of 1. Referring to the model, it means $\varphi$ equals 1; i.e. it is not possible (for A, or B, or C) to scale up their production without increasing input/cost). On the other hand, the efficiency score of D will be less than 1, and $\varphi$ takes a value greater than 1 (and consequently, $\frac{1}{\varphi}$ is less than 1). Indeed, the value of is measured as the ratio of OD divided by OB.

Note that the efficiency score is the value of the normalized composite index calculated using (i) outputs and inputs (or total cost) of each individual DMU and (ii) the set of weights produced by the linear programming in DEA for each DMU.

Figure S1: Frontier constructed using DEA technique: efficient vs. inefficient DMUs

O

A

B

C

D

Q1

Q2

$Q_{2B}$A

In the context of our proposed economic evaluation framework for data collection methods, we can apply the DEA methodology by establishing that:

- Each data collection method can be considered as a DMU. They produce the outputs/outcomes of “good vital events data”, which can be measured by the quality attributes (e.g. relevance, accuracy, and timeliness). The inputs for that production unit are in amount of inputs required to collect the relevant data by each method, for example, human resources, facilities, and equipment. These inputs can then be aggregated into a single item of input - “total cost”. Thus, we can view each data collection method as a multiple-output single-input DMU.
- DMU can be evaluated and ranked according to their productivity: that is, how much “good data” a method (i.e. production unit) can produce given the cost. Alternatively, we can think of this question as: “for a given level of quality data desired, how much money will we need to pay if a particular method is used?” Different data collection methods will have their own maximum productivity, that is, the highest possible quality standard it can achieve. Therefore, they can be ranked from best (most productive) to worst (least productive).
- We are interested in learning which methods of data collection outperform the rest, taking into account all the quality attributes and costs. Those methods that have the highest empirical productivity score are considered more “efficient” than other data collection methods. This implies that we want to measure the productivity and then the efficiency scores of all data collection methods to rank them from best (most efficient) to worst (least efficient).

Hence, the DEA will produce a cost-efficiency index that can be used for ranking. This index has a range of [0–1] and the closer the index to unity, the more cost efficient the DCM. A step-by-step summary of the estimation process is provided in Box S1.

| **Box S1:** Estimation Process | | |
| --- | --- | --- |
| **Stage** | **Steps** | **Description** |
| **1** | 1.1 | Collect information for each data collections: objectives, target population, actual coverage, data production and quality control |
|  | 1.2 | Decide on the DCMs to be included: |
|  |  | Two scenarios computed: 9 (excluding CRVS) or 10 (including CRVS) |
|  | 1.3 | Define output: we use ‘target population’ as the approximation for quantity of data |
|  |  |  |
| **2** | 2.1 | Assign scores for each quality criteria for each DCMs |
|  |  | Two scenarios computed: single score per criteria vs. panel of scores per criteria |
|  | 2.2 | Combine sub-indicators into five main indicators (using arithmetic average) - to reduce the data quality dimensions for the DEA analysis |
|  | 2.3 | Calculate composite quality index |
|  | 2.3a | Calculate unweighted quality index (QI) - arithmetic mean: using all sub-indicators |
|  | 2.3b | Calculate DEA-based quality index (QI) |
|  | 2.4 | Decide on the apportioning rule: decision based on (i) mortality share of the overall data collection objectives by the system; (ii) share of fixed cost (% adjusted down if large fix cost); (iii) sample size (% adjusted down if large sample size) |
|  | 2.5 | Collect cost information: |
|  |  | Collect the annualised total cost for each DCMs |
|  |  | Calculate the (annualised) total cost for vital statistic collection (out of the total cost for each DCMs) |
|  |  |  |
| **3** | 3.1 | Calculate the output: quality-adjusted target population (QADI) |
|  |  | QADI = composite quality index × target population |
|  | 3.2 | Calculate the cost per QADI |
|  |  | Cost per QADI = Total cost (for vital statistics) ÷ QADI |
|  | 3.3 | Use DEA to compute the cost-efficiency index: |
|  |  | Compute quality adjusted events for the five main quality index (representing five outputs - events adjusted for different quality dimensions), normalise them and the total cost for vital statistics |
|  | 3.4 | Rank DCMs by each set of scores using either: (i) Cost per QADI and (ii) Cost efficiency index |

# References

1. Drummond M, Sculpher M, Torrance G, O'Brien B, Stoddart G (2005) Methods for the economic evaluation of health care programmes. USA: Oxford University Press. 379 p.

2. Campbell H, Brown R (2004) Benefit cost analysis: financial and economic appraisal using spreadsheets.

3. Mahapatra P, Shibuya K, Lopez AD, Coullare F, Notzon FC, et al. (2007) Civil registration systems and vital statistics: successes and missed opportunities. The Lancet 370: 1653-1663.

4. Mathers CD, Fat DM, Inoue M, Rao C, Lopez AD (2005) Counting the dead and what they died from: an assessment of the global status of cause of death data. Bulletin of the World Health Organization 83: 171-177.

5. Muennig P (2008) Cost effectiveness analysis in health: A practical approach. San Francisco: Jossey-Bass. 266 p.

6. Coelli TJ, Rao DSP, O'Donnell CJ, Battese GE (2005) An introduction to efficiency and productivity analysis. New York: Springer.

7. Fried HO, Lovell CAK, Schmidt SS (2008) The measurement of productive efficiency and productivity growth. New York: Oxford University Press.

1. This variant of CEA is commonly referred to as cost utility analysis (CUA). CUA was developed to help decision-makers asses the value of alternative interventions that have very different health benefits. [↑](#footnote-ref-1)
2. Note that we specify the model with one input only because this applies directly to our evaluation framework. In other applications the model can be expanded to multiple inputs. [↑](#footnote-ref-2)
3. Note that we use only two outputs for the graphical illustration. Mathematically, the model can accommodate multiple-output and multiple-input cases just as well as the two-output one-input case. [↑](#footnote-ref-3)
